# The Remarkable Diversity of Vertebrate Bitter Taste Receptors: Recent Advances in Genomic and Functional Studies

**DOI:** 10.3390/ijms252312654

**Published:** 2024-11-25

**Authors:** Akihiro Itoigawa, Tomoya Nakagita, Yasuka Toda

**Affiliations:** 1 Department of Agricultural Chemistry, School of Agriculture, Meiji University, Kawasaki 214-8571, Kanagawa, Japan; 2 Japan Society for the Promotion of Science, Chiyoda-ku 102-0083, Tokyo, Japan

**Keywords:** bitter taste perception, G protein-coupled receptors, molecular evolution

## Abstract

Bitter taste perception is crucial for animal survival. By detecting potentially harmful substances, such as plant secondary metabolites, as bitter, animals can avoid ingesting toxic compounds. In vertebrates, this function is mediated by taste receptors type 2 (T2Rs), a family of G protein-coupled receptors (GPCRs) expressed on taste buds. Given their vital roles, T2Rs have undergone significant selective pressures throughout vertebrate evolution, leading to frequent gene duplications and deletions, functional changes, and intrapopulation differentiation across various lineages. Recent advancements in genomic and functional research have uncovered the repertoires and functions of bitter taste receptors in a wide range of vertebrate species, shedding light on their evolution in relation to dietary habits and other ecological factors. This review summarizes recent research on bitter taste receptors and explores the mechanisms driving the diversity of these receptors from the perspective of vertebrate ecology and evolution.

## 1. Introduction

Vertebrates use a variety of sensory systems, such as taste, smell, vision, touch, and hearing, to obtain the environmental cues essential for their survival. Among them, taste is crucial for evaluating the nutritional value and potential toxicity of food before ingestion. Different species rely on diverse food sources, resulting in significant variation in taste stimuli detection among species. Consequently, taste receptors exhibit remarkable diversity in gene repertoire, receptive breadth, and sensitivity to tastants, often correlating with an animal’s ecology, including habitat, dietary preferences, and feeding strategies. Among the basic tastes (sweet, umami, bitter, sour, and salty), the T2R receptor family, which is encoded by *Tas2r* genes, was identified as bitter taste receptors around the year 2000 [1,2,3]. Since then, the gene repertoires and functions of these receptors have been analyzed in various species. In particular, the sequencing of numerous vertebrate genomes over the past decade has significantly advanced the research on taste receptors in various non-model animals, including those that are difficult to access or endangered, thereby enhancing our understanding of the evolution and diversity of taste receptors in vertebrates. In this review, we highlight recent achievements in bitter taste receptor research from an ecological and evolutionary perspective, mainly focusing on taste function.

## 2. T2R Receptors: Phylogeny and Evolutionary Origins

**Figure 1:**
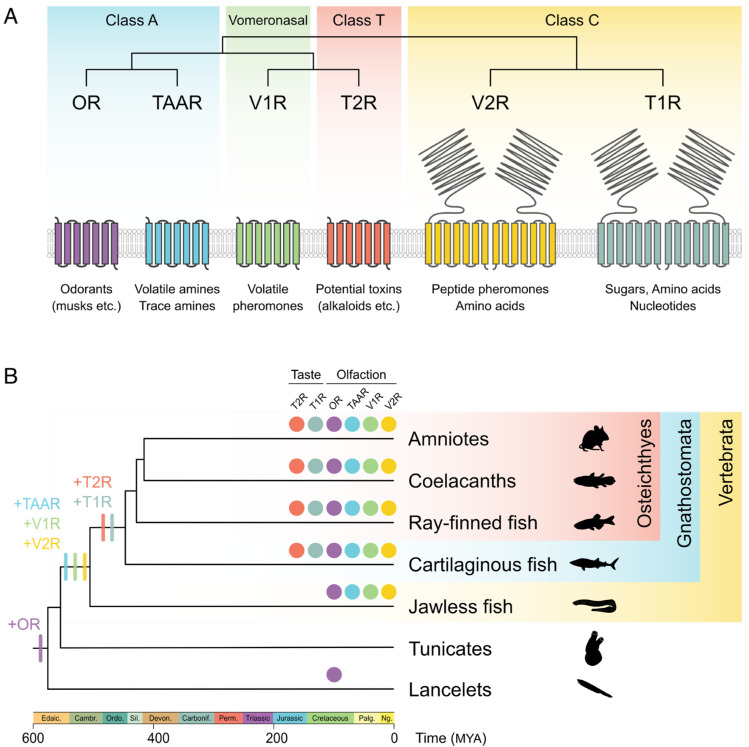
Classification and origins of T2Rs in chemosensory GPCR families. (**A**) A schematic illustration of the phylogeny, structures, and ligands of chemosensory GPCR families. The GPCR family classification and phylogeny refer to GPCRdb and several literature sources [22,23,24,25]. (**B**) The evolutionary origins of chemosensory GPCR families in Chordata. The animal silhouettes are retrieved from Phylopic (https://www.phylopic.org/, accessed on 28 May 2024).

Bitter taste is one of the chemical senses (taste and olfaction) and is considered to be an important sense for vertebrates to detect potential toxins in food, as all toxic compounds do not always exhibit bitterness [4]. Bitterness is mediated by a GPCR family member, T2Rs [1,2,3]. T2Rs are mainly expressed in taste bud cells on sensory organs, such as the tongue and oral palate, and detect potentially harmful substances with diverse structures [5,6]. Chemical senses other than bitterness are also mainly mediated by GPCRs similar to T2Rs. Vertebrates have six major GPCR families responsible for taste and olfaction (Figure 1A). Taste receptors include taste receptor type 1 (T1Rs), encoded by *Tas1r* genes, as well as T2Rs, while odorant receptors are classified into olfactory receptors (ORs), trace amine-associated receptors (TAARs), and vomeronasal receptor type 1 and type 2 (V1Rs and V2Rs, respectively). T2Rs detect potentially harmful substances, whereas T1Rs detect nutrient-related molecules such as sugars, amino acids, and nucleotides as sweet or umami taste. T2Rs have a small N-terminus extracellular domain similar to ORs, TAARs, and V1Rs, unlike T1Rs and V2Rs, and their phylogenetically closest relative is the V1R family (Figure 1A).

The evolutionary origins are different among chemosensory genes (Figure 1B). Odorant receptors have earlier origins than taste receptors. *OR* genes were already present in the common ancestor of Chordata, and the origins of other odorant receptor genes were traced back to the common ancestor of vertebrates [7,8,9,10]. Although *OR-like* genes, encoding class A GPCRs, are also reported in invertebrates such as Echinodermata (e.g., sea urchins and starfish) and Cnidaria (e.g., hydras and sea anemones), their orthology with vertebrate-type *ORs* is questionable [10,11,12,13,14,15]. In taste receptor genes, *Tas1rs* originated in the common ancestor of jawed vertebrates (Gnathostomata) [16,17], whereas it has been believed that the evolutionary origin of the *Tas2rs* traced back to the common ancestor of bony vertebrates (Osteichthyes), due to the absence of *Tas2rs* in cartilaginous fish (Chondrichthyes) and jawless fish (Cyclostomata) genomes [16,18]. However, several research groups have recently identified *Tas2rs* in a subclass of cartilaginous fish, Elasmobranchii (sharks, rays, skates, sawfish, and their close relatives) [8,19,20]. Consequently, the evolutionary origin of the *Tas2r* gene family is now assumed to be the common ancestor of jawed vertebrates, similar to the *Tas1r* gene family (Figure 1B) [8,17]. To date, no *Tas2r* orthologs have been found in jawless fish [8,20], although lampreys, a lineage of jawless fish, have a well-developed gustatory system that includes taste buds [21]. Thus, it remains an open question whether ancient jawless fish had *Tas2r* orthologs and subsequently lost them or whether they possess unknown genes that function as bitter receptor genes instead of *Tas2rs*.

## 3. Evolution of *Tas2r* Gene Repertoires

**Figure 2:**
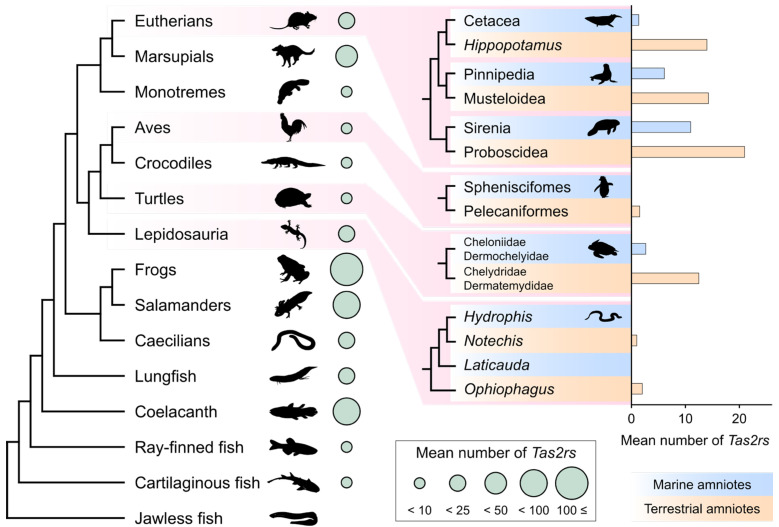
Variation of *Tas2r* gene number in vertebrates. The mean number of *Tas2rs* in major vertebrate lineages is represented as different sized circles. The inset bar plot shows that the comparisons of the mean number of *Tas2rs* between marine and terrestrial amniotes are represented as bar plots. Data were retrieved from Policarpo et al. (The number of complete *Tas2rs* with a predicted seven-transmembrane topology from the whole-genome assembly with > 80% BUSCO gene completeness) [8]. The animal silhouettes are obtained from PhyloPic (https://www.phylopic.org/, accessed on 28 May 2024). The silhouettes of the Tasmanian devil and platypus drawn by Sarah Werning are reused under the CC BY 3.0 (https://creativecommons.org/licenses/by/3.0/). The silhouette of *Hydrophis curtus* drawn by Christina Zdenek and the manatee drawn by Flurin Leugger are reused under CC BY 4.0 (https://creativecommons.org/licenses/by/4.0/).

**Figure 3:**
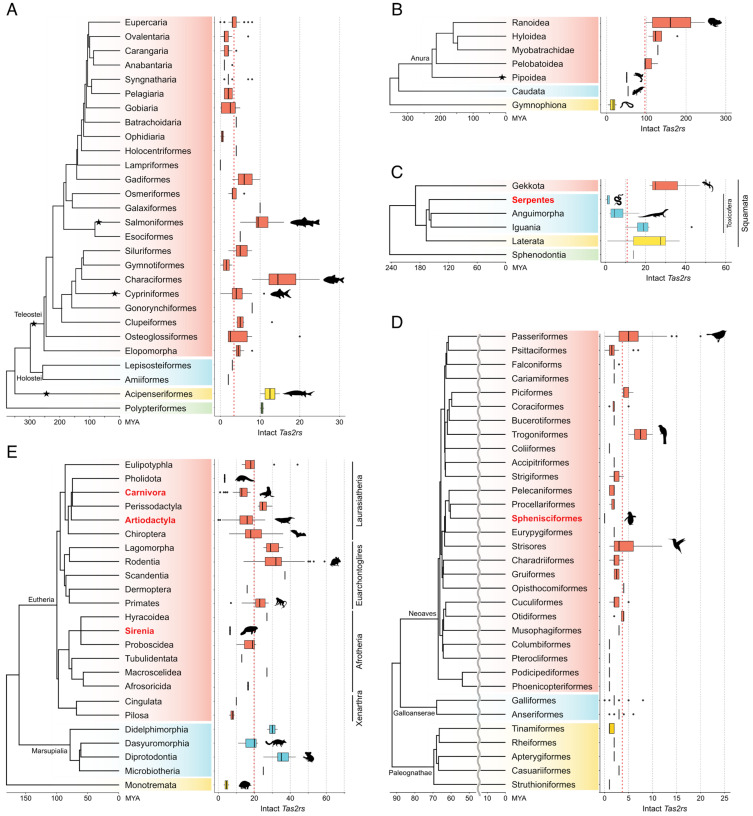
Variation of *Tas2r* gene number within representative vertebrate clades. The intact *Tas2r* gene number for (**A**) ray-finned fish, (**B**) amphibians, (**C**) birds, (**D**) Lepidosaurians, and (**E**) mammals is shown as boxplots with the mean *Tas2r* number for each clade. Phylogenetic trees were retrieved from previous studies (ray-finned fish, amphibians, and birds) [58,59,60] or the TimeTree 5 database (Lepidosaurians and mammals) [61]. Stars indicate whole-genome duplication events within the branches. Taxonomic groups in red include species fully adapted to aquatic environments. The taxonomic clades mentioned in the main text are marked with animal silhouettes. Animal silhouettes are obtained from PhyloPic (https://www.phylopic.org/, accessed on 28 May 2024). The silhouettes of the koala and numbat drawn by Sarah Werning and the silhouette of the vampire bat drawn by Roberto Díaz Sibaja are reused under CC BY 3.0 (https://creativecommons.org/licenses/by/3.0/). The silhouette of the manatee drawn by Flurin Leugger are reused under CC BY 4.0 (https://creativecommons.org/licenses/by/4.0/).

The evolution of the vertebrate *Tas2r* repertoire has been studied in a vast number of species (over 1500 species as of 2024) [8,19,20,26,27,28,29,30,31,32,33,34,35,36,37,38,39,40,41,42,43,44,45,46,47]. Generally, the receptors were identified from genome assemblies via BLAST-based gene mining. In many of these studies, intact genes were defined as those hit sequences that are predicted to encode proteins with a seven-transmembrane topology and form a monophyletic clade with known Tas2rs in a phylogenetic tree. This definition is primarily adopted in the present study. The number of *Tas2r* genes varies widely among vertebrates, ranging from zero (e.g., some cetaceans, penguins, and sea snakes) to over two hundred in certain frogs (Figure 2 and Figure 3) [8,32,41,42,45]. Cartilaginous fish and ray-finned fish generally have small *Tas2r* repertoires, whereas diversification and expansion of the gene family occurred in the lineage leading to tetrapods [8]. The *Tas2r* gene family has experienced drastic birth-and-death evolution [8,26,48]. Consequently, there are many lineage-specific *Tas2r* subtypes [8,26]. For example, there is no orthologous *Tas2rs* between “fish” (cartilaginous fish, ray-finned fish, and lobe-finned fish) and amniotes (mammals, reptiles, and birds), whereas some amphibians (e.g., salamanders and caecilians) have both amniote-specific *Tas2r* subtypes and *Tas2r* subtypes shared with coelacanths [8,28]. Although this relationship is supported by several studies [8,20,26,28], the unified classification of *Tas2r* subtypes across vertebrates and the phylogenetic relationships among the subtypes are not fully established. This is because the topology of the *Tas2r* gene tree is prone to change depending on the input dataset [8,20,28]. For example, the true topology near the stem root of the *Tas2r* gene tree [8,20,28] and whether there is a *Tas2r* subtype globally shared among ray-finned fish, lobe-finned fish, and amphibians [8,28] are topics for further research.

In vertebrates, a general trend shows that herbivores have larger *Tas2r* repertoires than carnivores [26]. The number of *Tas2rs* in amniotes is also influenced by feeding patterns and living environments. Species that swallow their food whole, having less opportunity to sense the chemicals from their prey, tend to have fewer *Tas2rs* [32,35,45,46]. The transition from land to marine environments also leads to a reduction in *Tas2r* repertoires, as observed in other chemoreceptor genes (see the inset of Figure 2) [8,32,44,45,46,49]. Furthermore, the number of *Tas2rs* is associated with other chemical sensing modalities [8]. Niimura et al. found that gains or losses of *Tas2rs* occurred synchronously with those of *ORs*, *V1Rs*, and *V2Rs* during Hystricomorph rodent evolution, suggesting no trade-off among different chemical sensing modalities [50]. It should be noted that such trends in the *Tas2r* repertoire related to vertebrate ecology or phenotypes are not consistently observed across all vertebrate classes or orders. Therefore, it is crucial to identify the specific periods in phylogenetic trees when *Tas2r* genes were gained or lost and to individually estimate the causes of these changes [8,29,41,50]. Hereafter, we overview the characteristics of the receptor repertoire in each vertebrate lineage.

### 3.1. Cartilaginous, Ray-Finned, and Lobe-Finned Fish

Cartilaginous fish have the smallest repertoires (ranging from zero to one) among vertebrates. Their *Tas2rs* consist of a single, unique ortholog, which is considered one of the earliest-emerged *Tas2r* orthologs [8,19,20]. This gene is highly conserved with a low frequency of gene duplication, and its presence or absence is not correlated with diet [20].

Ray-finned fish also have small repertoires, not correlated with any ecological traits such as diet, temperature preference, water habitat, or living depth [8]. Ray-finned fish receptors are classified into three or four clades, with varying degrees of gene gains or losses between clades [8,36]. Some lineages have experienced massive expansion of *Tas2rs* (Figure 3A). In particular, Characiformes has one of the largest *Tas2r* repertoires [8]. For example, the Mexican tetra (*Astyanax mexicanus*) has over twenty *Tas2rs*, roughly the same number as humans [8,36]. This species has two morphs: cave and surface forms. Cave populations have an increased number of taste buds, enhanced chemosensory capabilities, and improved food detection in dark environments [51,52]. The cave form has 21 *Tas2rs*, whereas the surface form has 25 [8,36]. Given that gene mining methodologies and genome assembly quality affect gene identification, further detailed investigation using the same gene mining method using high-quality genome assembly of two morphs and population data is required to understand the differences in bitter taste between two morphs. For additional details on this species’ taste study, please refer to another review [51]. Ray-finned fish have experienced lineage-specific whole-genome duplication (WGD) events in a few lineages, including Acipenseriformes (sturgeons and paddlefish), the stem of Teleostei, Salmoniformes, and carps (a subclade of Cypriniformes). Since carps, Salmoniformes, and Acipenseriformes have a relatively larger number of *Tas2rs* (Figure 3A), WGD might be a factor affecting *Tas2r* numbers in ray-finned fish.

The *Tas2r* repertoire of lobe-finned fish differs largely between coelacanths (Coelacanthiformes) and lungfish (Dipnoi). The West Indian Ocean coelacanth (*Latimeria chalumnae*) has the second largest *Tas2r* repertoire among vertebrates, following batrachians (frogs and salamanders) [8,28,37,38]. Coelacanth *Tas2rs* expanded through species-specific gene duplication, likely due to accumulated repeat elements surrounding *Tas2r* loci [37]. Of the coelacanth *Tas2rs*, *Tas2r01* is orthologous to ray-finned fish *Tas2r1*, the only known clear orthologous gene pair between the ray-finned fish and lobe-finned lineages [8,28,37]. This gene is believed to be one of the basal *Tas2r* orthologs. Conversely, lungfish have less than half the *Tas2rs* of the coelacanth [8]. Since both lungfish and tetrapods lack *Tas2rs* orthologous to coelacanth *Tas2r01*, this ortholog was likely lost in the common ancestor of lungfish and tetrapods.

### 3.2. Amphibians

Amphibians exhibit remarkable variations in *Tas2r* gene repertoires (Figure 2 and Figure 3B). Batrachians have the largest repertoires among vertebrates, with the Japanese wrinkled frog (*Glandirana rugosa*) having 247 *Tas2rs* [8,41,42]. In contrast, caecilians (Gymnophiona) have relatively smaller repertoires than batrachians, ranging from 3 to 25 [8,41]. Many caecilians, unlike batrachians, are highly adapted to subterranean environments and are carnivorous, likely leading to reduced exposure to potential toxins and thus a reduced importance of bitter taste [41]. Anuran (frog) *Tas2rs* frequently expand in a lineage-specific manner [41]. In some frogs, many clusters of *Tas2rs* are observed on certain chromosomes, with particularly large clusters tending to be located near chromosome ends [41]. On the other hand, allotetraploid *Xenopus* frogs (African clawed frog, *X. laevis*, and Marsabit clawed frog, *X. borealis*) have almost the same number of *Tas2rs* as their diploid relative, the tropical clawed frog (*X. tropicalis*) [8,41]. Given these pieces of evidence, one of the most likely mechanisms for the massive expansion of batrachian *Tas2rs* is tandem duplication near chromosome ends, where nonhomologous recombination frequently occurs, rather than large-scale mutations such as WGD. It is so far unclear whether repeat elements are involved in *Tas2r* duplication of batrachians, as in the coelacanth [37]. To understand the expansion mechanisms in more detail, a larger comparative genomic analysis between batrachians and other vertebrates is required. Furthermore, the ecological and physiological significance of batrachian *Tas2r* expansion also remains controversial. Many batrachians are sit-and-wait or ambush predators, suggesting the biological importance of bitter taste lies more in the ability to detect and reject inedible or potentially harmful prey once captured rather than in detecting prey [53]. Additionally, many batrachians inhabit both aquatic and terrestrial environments, leading to encounters with a larger variety of harmful substances. Anurans, in particular, experience an ontogenetic dietary shift from herbivory or detritivory to carnivory or insectivory through metamorphosis. Hao et al. demonstrated that a subset of *Tas2rs* is differentially expressed in the oral cavity between tadpole and adult American bullfrogs (*Lithobates catesbeianus*), suggesting differential use of *Tas2rs* between life stages [42]. However, only a fraction of 180 bullfrog *Tas2rs* show differential expression in the oral cavity during metamorphosis. Therefore, there may be other potential drivers for *Tas2r* expansion beyond taste-related functions, such as extra-oral roles.

### 3.3. Birds and Reptiles

Avian *Tas2r* repertoires are generally small, with some lineages, such as Passeriformes (perching birds), Trogoniformes (trogons), and Strisores (nighthawk, sparrows, and hummingbirds), experiencing significant expansions of *Tas2rs*, up to 20 genes (Figure 2 and Figure 3D) [8]. Conversely, Sphenisciformes (penguins), which lack taste buds on their tongues, have lost all *Tas2rs* [8,39,45]. Possible causes of this loss include swallowing food whole and marine adaptation (both interrelated), while the previously suggested association with their life in cold environments is questionable, as it has not been observed in other cold-adapted birds and mammals [8,45]. The number of intact *Tas2rs* correlates with dietary preference in birds; herbivorous and insectivorous species have more *Tas2rs* than carnivorous species [39]. A recent large-scale analysis indicated a correlation between *Tas2r* number and migratory behavior, though the dietary association was not observed in that study [8].

Non-avian reptiles, including turtles, crocodilians, lizards, and snakes, generally have small *Tas2r* repertoires, except for some lizards (Figure 2 and Figure 3C). The number of *Tas2rs* in Lepidosauria (lizards and snakes) is highly variable (Figure 3C). Many lizards possess more than twenty *Tas2rs*, with the Japanese gecko (*Gekko japonicus*; Gekkota) having nearly 50 *Tas2rs* [8,35]. In contrast, snakes (Serpentes) and *Varanus* monitor lizards (Anguimorpha), which have taste-bud-less tongues, have a minimal *Tas2r* repertoire, ranging from zero to three [8,35,40]. In squamate reptiles, herbivorous and insectivorous species have more *Tas2rs* than carnivorous species, indicating dietary association [35]. Additionally, foraging patterns may influence *Tas2r* repertoires [35]. Snakes and monitor lizards, which swallow their prey whole, have small *Tas2r* repertoires similar to cetaceans and penguins (though marine adaptation may also play a role) [35,44,45,46]. Sea turtles and sea snakes have reduced *Tas2r* repertoires compared to their terrestrial relatives, exemplifying genetic convergence relevant to marine adaptation (see the inset of Figure 2) [8]. Notably, sea snakes have completely lost the few remaining *Tas2rs* found in terrestrial snakes [8], suggesting that both swallowing feeding and marine adaptation contribute to the reduction of *Tas2r* repertoires in snakes. This raises the possibility that for most terrestrial snakes, the few remaining *Tas2rs* may be crucial for terrestrial life.

### 3.4. Mammals

Mammals have medium-sized *Tas2r* repertoires, ranging from 0 (some cetaceans) to 59 (African woodland thicket rat, *Grammomys surdaster*) (Figure 2 and Figure 3E) [8,26,29,32,50]. Monotremes (platypus and echidna) have one of the smallest numbers of *Tas2rs* in non-fully aquatic mammals, whereas therians (eutherians and marsupials) have an average of ten or more. In marsupials, Diprotodontia (e.g., koalas, possums, and kangaroos) have the largest number of *Tas2rs*, followed by Didelphimorphia (opossums), Microbiotheria (monito del monte), and Dasyuromorphia (e.g., quolls and Tasmanian devils). In eutherians, Euarchontoglires (e.g., rodents, rabbits, and primates) have the largest *Tas2r* repertoire, followed by Afrotheria (e.g., elephants and tenrecs) and Laurasiatheria (e.g., ruminants, bats, and Carnivora). Xenarthra (e.g., sloths and armadillos) have the smallest *Tas2r* repertoire, 10 or fewer genes, among four major eutherian lineages. A recent large-scale study showed that the mammalian *Tas2r* repertoire is larger in omnivorous species, followed by herbivorous and carnivorous species [8]. This trend differs from the vertebrate-wide trend inferred in the earlier study [26], although in a mammalian lineage, Laurasiatheria, the trend matches the vertebrate-wide pattern [32]. Swallowing behavior is also associated with *Tas2r* reduction in mammals, such as cetaceans and pinnipeds [32].

Massive reductions in *Tas2r* repertoires have independently occurred in many mammalian lineages (Figure 3E). For example, pangolins (Pholidota), a Laurasiatherian lineage, has only a few *Tas2rs*, likely due to dietary specialization to ants and termites (myrmecophagy) [8,32]. Similar reductions occurred convergently in marsupial and monotreme myrmecophagous species, like the numbat (*Myrmecobius fasciatus*; Dasyuromorphia) and the short-beaked echidna (*Tachyglossus aculeatus*) [43,54]. Sanguivorous vampire bats also have reduced *Tas2rs* [55,56]. Colobine monkeys, specialized folivores, reduced the number of *Tas2rs* after diverging from their omnivorous cercopithecine relatives, which is thought to be associated with high tolerance to toxic compounds through mechanisms like foregut fermentation by symbiotic microbiota [47]. In relation to marine adaptation, convergent reductions in *Tas2r* repertoires are also found in marine mammals, including sirenians, pinnipeds, and cetaceans (see the inset of Figure 2) [8,32,44,46,49]. Conversely, massive expansions of receptors are also found in many mammalian lineages (Figure 3E). For instance, the koala (*Phascolarctos cinereus*) has a large *Tas2r* repertoire including tandemly duplicated *Tas2r41* and *Tas2r705* [31,57]. Some of these duplicates are sensitive to analogs of eucalyptus toxins, potentially aiding in the detection of eucalyptus toxins and the selection of edible eucalyptus leaves [31,57]. In primates, the common ancestor of hominoids and cercopithecoids independently experienced a massive expansion of *Tas2rs*, possibly related to increased folivory with increased body size (i.e., switching of protein sources from insects to leaves) [29].

## 4. Functional Evolution of T2Rs

### 4.1. Ancestral Functions of T2Rs

The frequent expansion and contraction of *Tas2rs* and the great diversity of agonist repertoires make it challenging to identify the ancestral function of T2Rs. However, the early-emerged orthologs or functionally conserved orthologs between distantly related species provide insight into ancestral T2R functions. For instance, a distinct one-to-one ortholog conserved between lobe-finned and ray-finned lineages, namely ray-finned fish T2R1 and coelacanth T2R01, have the same agonists, chloroquine and denatonium benzoate [28]. Cartilaginous fish-specific T2R orthologs also respond to these substances, suggesting that these agonists may be reflected in the function of T2R founders [19,20]. Additionally, endogenous steroids, such as bile acids and steroid hormones, are known as agonists of T2Rs in diverse vertebrates from ray-finned fish to mammals [28,62,63]. Shark receptors also recognize bile acids, suggesting the bifunctional nature of T2R founders to endogenous and exogenous compounds [19].

### 4.2. Relationships Between the Tuning Breadth and Repertoire Size of T2Rs

**Figure 4:**
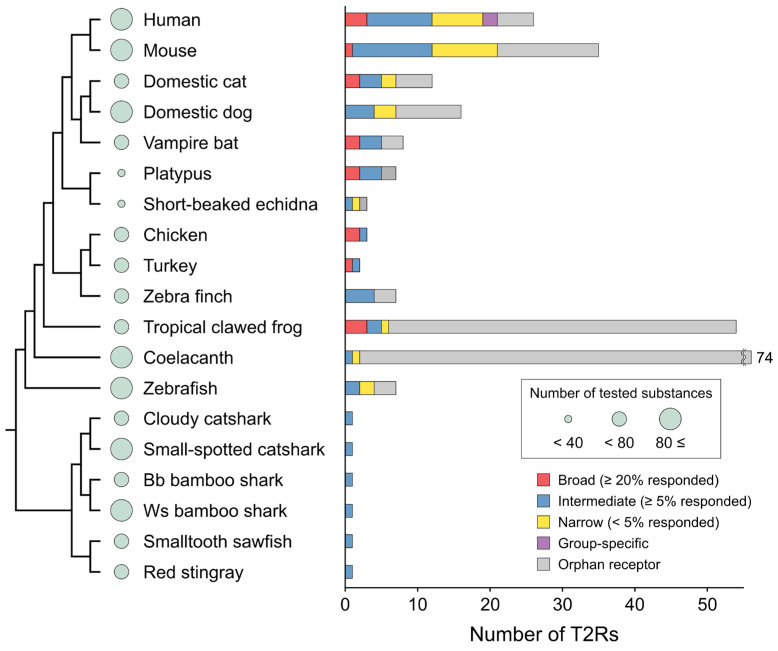
The tuning breadth of vertebrate T2Rs. The receptive ranges of representative vertebrate T2Rs are classified into “broad”, “intermediate”, “narrow”, and “group-specific” based on the ratio of agonists in the total number of substances tested in the screening studies, which included in total over 20 substances except for a receptor of vampire bats (19 substances) [5,6,19,20,27,28,56,57,64,65,66,67,73] and represented in bar plots. The number of tested substances is shown as the size of circles next to the species names. Bb bamboo shark and Ws bamboo shark indicate the brownbanded bamboo shark and whitespotted bamboo shark, respectively.

T2Rs from many vertebrates have been functionally characterized using bitter compound panels [5,6,19,20,27,28,30,42,56,57,64,65,66,67,68,69,70] (see the BitterDB database (https://bitterdb.agri.huji.ac.il/dbbitter.php) [71,72] for humans, mice, cats, and chickens and Supplementary Table S1 for other species). These data on the tuning breadth of receptors indicate an intriguing relationship with *Tas2r* repertoire size (Figure 4). Even species with fewer receptors, such as chickens and turkeys, have broadly tuned receptors, suggesting a wider receptive range than expected from the size of *Tas2r* repertoires [27,28]. Conversely, the receptive range in the dietary specialized echidna is narrower than in the platypus, aligning with a reduction in the *Tas2r* repertoire [57]. Species with many receptors, such as humans and mice, also have a few broadly tuned receptors, while many receptors are intermediate or narrowly tuned [73]. Consequently, the size of the *Tas2r* repertoire does not always reflect the receptive range of bitter substances. To fully understand the animals’ bitter taste space, analyses of both receptor repertoire and tuning breadth are essential. However, it should be noted that bitter compound panels used in most studies are biased toward compounds known as bitter to humans. Further investigation with panels of ecologically relevant substances, such as defense substances of potential prey, would be worthwhile to examine the significance of bitter sense in each animal.

### 4.3. Functional Diversity of Orthologous T2Rs Among Species

The interspecific variation of T2R properties is observed in ligand sensitivity and specificity, and receptive ranges. Comprehensive comparisons between human and mouse T2Rs revealed that even one-to-one orthologs generally have distinct agonist profiles [6]. Similarly, T2R1 has achieved differential agonist spectra among bats [69]. In contrast, there is also evidence for the conservative ligand specificity of T2Rs among distantly related species. For instance, eutherian T2R16 and its marsupial ortholog (T2R705) respond to β-glucosides and their analogs, one of the major plant secondary metabolites, with high specificity in many species [5,30,57,74,75,76,77]. Furthermore, monotreme T2Rs in the same orthologous group as T2R16/T2R705 respond to β-glucosides, suggesting this bitter sensing trait was retained from the common ancestor of mammals [57]. Sensitivity to metal ions in T2R7 is conserved between humans and vampire bats [70,78,79], and zebrafish T2R1 and its coelacanth ortholog T2R01 have the same agonist property mentioned above [28].

The functional divergence in agonist sensitivity of each T2R receptor contributes to species-specific bitter taste space. For example, T2R16 orthologs are known as receptors for β-glucosides and their analogs, but agonist sensitivity and selectivity frequently change. Reduced sensitivity to β-glucosides is independently observed in many primate lineages, with complete loss in marmosets, tamarins, and galagos [74,75,80]. In some lemurs, specific β-glucosides, that act as agonists in other primates, act as inverse agonists [77]. Behavioral tests corroborate some of these functional reductions, such as in macaques and lemurs [74,77]. Moreover, bamboo lemurs (*Hapalemur* and *Prolemur*), which rely on cyanogenic bamboo, have receptors that are less sensitive to β-glucosides [80]. This reduced sensitivity is inferred to be an advantageous trait for ingesting bamboo containing cyanogenic glucosides [80]. Conversely, enhanced sensitivity to β-glucosides is observed in some primates, such as the white-faced saki (*Pithecia pithecia*) and certain human haplotypes, which independently increased sensitivity to β-glucosides through the convergent mutation at the same amino acid position [75]. The T2R38 ortholog known as a receptor for phenylthiocarbamide (PTC), also exhibits functional differences between species. T2R38 orthologs respond to PTC in humans and domestic cats, but not in mice and domestic dogs [6,64,65,81]. Even within primates, humans, chimpanzees, and macaques have PTC-sensitive T2R38 [81,82,83], whereas folivorous colobine monkeys lost or reduced the PTC sensitivity of T2R38 through lineage-specific mutations [84,85]. This receptor phenotype corresponds to behavioral avoidance of PTC, hypothesized as an adaptation related to leaf-eating behavior [84,85].

Gene duplication is another major mechanism for functional divergence in T2Rs. Studies of highly duplicated T2Rs in hummingbirds (T2R1) and *Myotis* bats (T2R16) have demonstrated functional divergence among duplicated T2Rs [30,68]. Some receptor duplicates retain agonists shared with the no-duplicated ortholog in close relatives and acquire novel agonists, while others lose these shared agonists. This diversification may be related to the hummingbirds’ unique nectar diet and the ability of *Myotis* species to adapt to diverse environments across all continents except Antarctica [30,68].

Besides interspecific comparisons of T2R orthologs, ancestral sequence reconstruction is a useful approach to identify the key residues and evolutionary trajectory of receptor function. Studies on platyrrhines and strepsirrhines identified the evolutionary time points of functional changes by comparisons between extant species receptors and reconstructed ancestral receptors [80,86]. Reconstruction of intact sequences of pseudogenized T2Rs in modern humans revealed that functional redundancy could cause pseudogenization of receptors [87]. Because T2Rs form a multigene family, estimating accurate ancestral sequences prior to massive gene duplications can be challenging, but ancestral reconstruction is a powerful tool for understanding the evolutionary history of individual receptors at the functional level.

## 5. Intraspecific Variation in T2Rs and Agonist Sensitivity

Intraspecific variations of *Tas2r* repertoires and agonist sensitivity provide individual differences in bitter perception. These variations are numerous in human populations and can result in phenotypic differences in bitter perception [81,88,89,90]. It has long been debated how this diversity in human *TAS2Rs* was formed, with several probable causes, such as natural selection or demographic history being suggested [88,91,92,93,94,95,96]. A recent comprehensive genomic survey, using data from the 1000 Genomes Project, demonstrated that selective pressure on most *TAS2Rs* has relaxed during recent human evolution [97]. Genetic variations in *Tas2rs* are also observed in non-human animals, influencing taste perception or food selection [83,98,99,100,101,102,103,104]. For example, the polymorphism of three linked amino acid positions (P49A, A262V, and V296I) in T2R38 determines whether it is PTC-sensitive (PAV) or not (AVI) in humans [81,105]. Different polymorphic sites lead to similar phenotypic variations in non-human primates. Haplotypes with a loss of start codon are found in chimpanzees and Japanese macaques, while haplotypes with a premature stop codon or polymorphic sites leading to a loss of PTC sensitivity are present in several species of Sulawesi macaques [82,83,99,106]. These genetic variations correspond well to differences in aversive behavior toward PTC. T2Rs are also functionally differentiated among wild populations. In neighboring populations of the blind mole rat (*Spalax galili*) inhabiting contrasting soil environments (basalt and chalk), several T2R haplotypes enriched in the basalt population have a higher sensitivity to bitterants than those enriched in the chalk population [103]. This suggests that functional differentiation of T2Rs could aid in optimal food selection from the different food resources [103]. In giant pandas, the functional differentiation of T2R20, a quercitrin receptor, is associated with different quercitrin contents in dietary bamboo among populations [104]. In chimpanzees, most *Tas2r* haplotypes are specific to each of four subspecies, potentially linked to subspecies-specific dietary repertoires [98,107]. Further studies are needed to clarify the relationship between the functional differentiation of bitter taste receptors and diet in wild animal populations, as research on this topic is less extensive compared to human populations.

## 6. Extra-Oral Expressions of T2Rs in Vertebrates

### 6.1. Diverse Functions of Extra-Oral T2Rs

Although T2R expression outside oral tissues was already known in one of the earliest studies [3], subsequent studies have revealed that T2R expression is widely distributed in various extra-oral tissues such as the brain, muscles, cardiovascular system, skin, adipose tissues, immune cells, respiratory tract, gastrointestinal tract, and reproductive organs [108,109,110,111]. Human and rodent studies have revealed that the extra-oral receptors act as local chemoreceptors and are involved in important physiological functions, including not only protection from pathogens and harmful substances similar to their role in the oral cavity but also in metabolic regulatory pathways and the reproductive system.

For instance, T2Rs in the cardiac muscles and smooth muscles of various organs, such as the airway, gastrointestinal tract, vessel, uterus, and bladder, are involved in muscular relaxation (and contraction in some organs) [112,113,114,115,116,117,118,119,120,121,122,123]. In the respiratory tract, nasal tuft cells (known as solitary chemosensory cells) [124,125,126,127,128,129], tracheal tuft cells (known as brush cells) [130,131,132,133], and airway ciliated cells [126,134,135,136,137,138] have T2Rs responding to bitterants and/or bacterial quorum-sensing molecules (QSMs). These receptors induce bacterial clearance systems such as neurogenic inflammation, the production of antimicrobial molecules (nitric oxide and β-defensins), and the upregulation of mucociliary clearance [130,131,132,133,139,140,141]. In the gastrointestinal tract, T2Rs are found in specific epithelial cells, including enteroendocrine cells (EECs), tuft cells, goblet cells, and Paneth cells [142,143,144,145,146,147,148,149,150]. EEC T2R signals can modulate the secretion of distinct gut hormones (ghrelin, cholecystokinin, glucagon-like peptide-1, etc.) depending on the gut segments or cell types [150,151,152,153,154,155,156,157,158,159,160,161,162,163,164,165], which regulate food digestion, nutrient absorption, and metabolic homeostasis. Analogous to respiratory tracts, mouse T2Rs in intestinal tuft cells initiate a type 2 innate immune response to helminth infection [166]. Human T2Rs in goblet and Paneth cells can regulate the mRNA expression of antimicrobial peptides, mucins, and chemokines, potentially maintaining intestinal homeostasis [167]. Interestingly, T2Rs are also found in a variety of immune cells (e.g., lymphocytes, myeloid cells, neutrophils, leucocytes, and macrophages) as well as epithelial immune-related cells in the airways and intestines [168,169,170,171,172,173]. T2Rs detect QSMs in human macrophages, leading to enhanced bacterial phagocytosis [172,174]. Furthermore, T2Rs are also expressed in both male and female reproductive organs, such as the testis, sperm, and ovary, in humans and mice [116,175,176,177,178,179,180,181,182,183,184]. Ablation of testicular T2R105-expressing cells leads to loss of spermatids and infertility in mice [176]. Sperm T2Rs may be involved in chemotaxis by detection of progesterone or chemokines derived from cumulus–oocyte complex because progesterone is a T2R agonist [6,177,184]. Ovarian T2Rs may be involved in progesterone production [185].

T2R-expressing cell types, T2R subtypes, and functions in extra-oral tissues of humans and rodents are summarized in the tables of several recent review articles [109,110,111,186]. Please see the recent specific reviews for more details on the extra-oral functions of T2Rs and their inter- and intra-cellular mechanisms (e.g., overall review [109,174], smooth muscle and cardiovascular system [186,187], respiratory tract [124,139], gastrointestinal tract [111,151,188], and reproductive system [184,189]).

### 6.2. Interspecific Differences of Extra-Oral T2Rs

The recent research advances in extra-oral T2R functions are illuminating the broad roles in mammalian physiology. Many studies have demonstrated that stimulation of T2Rs by certain bitterants, such as denatonium benzoate, elicits similar physiological responses in a variety of organs between humans and rodents, whereas the chemical response properties of T2R-expressing cells may have species differences. This is because the receptor repertoire, tuning breadth, and agonist sensitivity are largely different between species. Indeed, comparing *Tas2r* expression in the gastrointestinal tract and muscle between humans and rodents, the expression patterns are often different even between one-to-one orthologs [151,187]. In the upper airways, QSMs, particularly acyl-homoserine lactones, can stimulate mouse tuft cells but do not appear to stimulate human cells [124]. These differences may be associated with species-specific habitats because the respiratory and gastrointestinal tracts are interfaces to external environments; hence, it is worthwhile to study them in the framework of evolution and environmental adaptation. In particular, it is important to study non-human primates, which have a similar *Tas2r* repertoire to humans, to clarify the specificities and evolution of humans and for application to human clinical practice. For instance, although not an exact match, the overall expression pattern of intestinal *Tas2rs* is very similar between humans and macaque monkeys [147,148], and further studies both in vivo and in vitro (primary or organoid culture) are expected in the future. It is also interesting to note that easily evolved T2Rs are used in internal organs that are less directly exposed to external stimuli, such as the brain, ovary, and muscle. Given the possibility that vertebrate T2Rs have potentially endogenous agonists, such as bioactive steroids [6,19,28,62,63], these internal functions may be important to understand the evolutionary ancestry of T2Rs.

Although the various physiological roles of extra-oral T2Rs have been identified in model rodents and humans, the functional conservation and diversity of extra-oral T2Rs across vertebrates remain poorly understood. This is because novel functions have recently been elucidated in rodents and humans, while research on non-mammalian vertebrates remains limited. Despite the limited number of studies, the expression of *Tas2rs* is observed in a variety of extra-oral tissues of cartilaginous fish, ray-finned fish, and birds [66,190,191,192,193,194,195]. This suggests that the extra-oral function of T2Rs developed as early as their oral function. However, it is so far difficult to compare gene expression patterns and putative functions among species because of the limited number of analyzed organs and few cellular level data. Even in zebrafish, the *Tas2r* expression across the entire body has not been fully analyzed; thus, it is an important direction to accumulate comparative transcriptomic information focusing on taste receptors and their signaling molecules, including other model animals (e.g., medaka fish and *Xenopus* frogs). Furthermore, CRISPR/Cas-based gene editing would enable in vivo analyses of the physiological functions of extra-oral taste receptors even in non-model animals, which will accelerate our understanding of evolution in extra-oral T2R physiology.

## 7. Structural Features of T2Rs in the GPCR Superfamily

**Figure 5:**
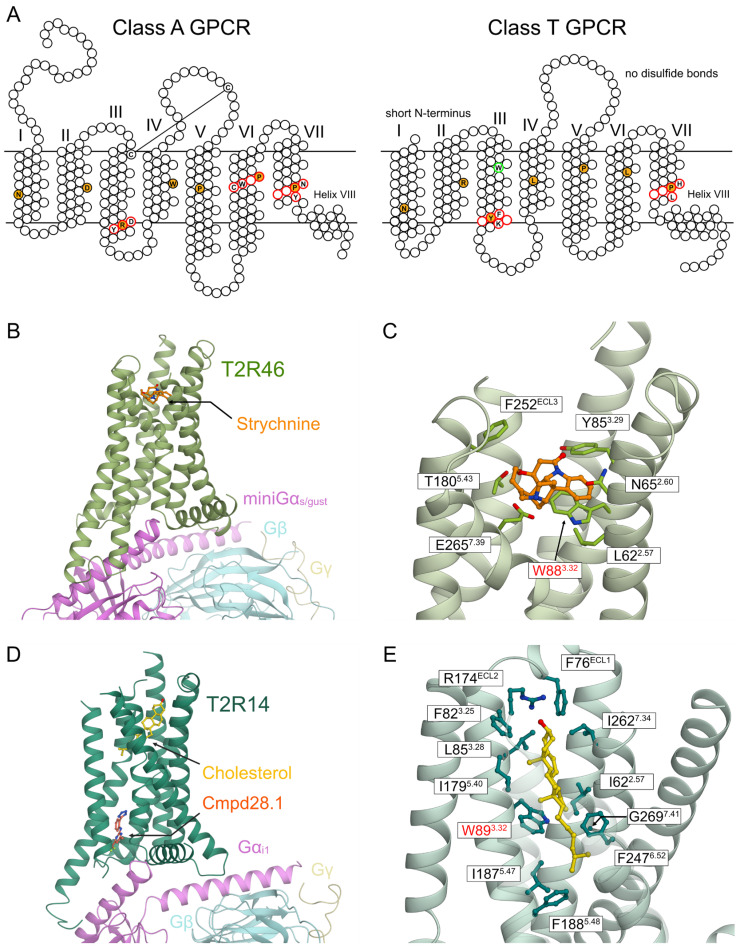
Three-dimensional structures of T2Rs. (**A**) The snake plots of class A and T GPCRs. The residues filled by orange indicate X.50 residues (generic residue numbering), most conserved residues of each transmembrane domain for each GPCR class. Residues colored in red and green indicate conserved sequence motifs and a key residue for ligand binding, respectively. (**B**) The overall structure of T2R46 complexed with strychnine retrieved from the PDB (7XP6). (**C**) The binding mode of strychnine to T2R46 retrieved from the PDB (7XP6). (**D**) The overall structure of T2R14 complexed with cholesterol and cmpd28.1 (an analog of fulfenamic acid) retrieved from the PDB (8VY7). (**E**) The binding mode of cholesterol to T2R14 retrieved from the PDB (8VY7). The illustrations were drawn using CueMol2 (http://www.cuemol.org/, downloaded on 24 May 2024).

In recent years, significant progress has also been achieved in the structural biology of bitter taste receptors. T2Rs have the typical GPCR motif; seven transmembrane domains with an extracellular N-terminus and an intracellular C-terminus. GPCRs are generally categorized into class A, B, C, and F depending on the shape of the N-terminus domain [1,2,3,196]. While T2Rs were previously classified as class A GPCRs due to their lack of an extracellular N-terminus domain, their unique amino acid sequences distinguish them from this class. Thus, the T2R family was classified into a distinct GPCR class, namely class T, in 2019 [197]. T2Rs differ from class A GPCRs in two major structural features (Figure 5A). First, the N-termini of most T2Rs are very short or almost nonexistent. This characteristic often makes it difficult to properly express T2Rs in a cell culture system; hence, N-terminus signal sequences of other membrane proteins such as rat somatostatin receptor 3 are often added to the native T2Rs to improve cell surface expression in vitro [198,199]. Second, the T2R family lacks a disulfide bond between transmembrane (TM) III and extracellular loop 2. The disulfide bond contributes to receptor stability and limits the width or variety of the ligand binding pocket; hence, this feature may allow the binding pocket of T2Rs to flexibly accept a variety of substances.

For a comparison of mutational effects, ligand interactions, and structural motifs, the transmembrane residues of GPCRs are numbered based on a specific numbering system. In Figure 5A, residues filled in orange represent X.50 residue based on the generic residue numbering in which the most conserved residue in each TM helix is numbered as 50 [200]. For instance, N^1.50^ in the class A GPCR indicates the most conserved residue in TM I. Furthermore, each class of GPCRs has well-conserved sequence motifs (Figure 5A). For example, class A GPCRs have motifs such as D^3.49^R^3.50^Y^3.51^, C^6.47^W^6.48^xP^6.50^, and N^7.49^P^7.50^xxY^7.53^ [201]. In contrast, T2Rs have activation-related motifs, F^3.49^Y^3.50^xxK^3.53^ and H^7.49^S/P^7.50^xxL^7.53^, which correspond to the class A motifs, D^3.49^R^3.50^Y^3.51^ and N^7.49^P^7.50^xxY^7.53^, respectively [201].

In 2022, Xu et al. reported the first experimental structure of human T2R46 complexed with and without strychnine by cryo-electron microscopy (cryo-EM) technology (Figure 5B,C) [202]. Furthermore, cryo-EM structures of human T2R14 complexed with cholesterol, flufenamic acid, and aristolochic acid were reported in 2024 (Figure 5D,E) [203,204,205]. These studies revealed the distinct 3D structures of T2Rs compared to any other GPCRs. The primary (orthosteric) binding site of T2Rs is located at the extracellular side of the transmembrane domain. The highly conserved tryptophan residue at position 3.32 (found in 84% of human T2Rs, shown as a green circle in Figure 5A), which is in the orthosteric binding site and forms CH−π or π−π interactions with ligands, plays a critical role in ligand binding for T2R14 and T2R46 (Figure 5C,E) [202,203,204,205]. As W^3.32^ also largely affects agonist sensitivity in the other T2Rs [206,207], it may generally contribute to the ligand binding of T2Rs by the same mechanism as T2R14 and T2R46. The experimental structure of T2R14 complexed with ligands demonstrates that this receptor has two or three distinct binding sites (Figure 5D) [203,204,205]. The most extracellular site is the orthosteric binding site, whereas the other intracellular sites are the allosteric binding sites. In particular, the molecule bound in the intracellular part interacts with the coupled G protein alpha subunit. These multiple binding sites may contribute to the broad receptive breadth range in T2R14. T2R14 and T2R46 are broadly tuned T2Rs; therefore, the experimental structures of narrowly tuned or group-specific T2Rs, such as T2R38 or T2R16, would be aid in understanding the mechanisms regulating the receptive ranges of ligands.

## 8. Conclusions

Recent advances in genomic and functional studies of taste receptors have highlighted the remarkable diversity of vertebrate bitter taste receptors. Both gene repertoires and protein functions are associated with vertebrate diets, although not always observed in all vertebrate taxa. In particular, many examples of such associations have been found in mammals and birds. Therefore, cooperation is required between sequence-based and protein function-based analyses to deeply understand the evolution of taste receptor functions associated with dietary variation. To identify the taste evolution involved in phenotypic and physiological changes in vertebrate evolution, it is also essential to test various species with appropriate outgroups (and sometimes the reconstructed ancestral sequences) and identify the key amino acid changes on the phylogenetic tree with high temporal resolution. Moreover, deeply understanding the genetic basis of dietary shift requires exploring the evolutionary coordination of taste receptors with other diet-related proteins, such as digestive/detoxification enzymes.

While comparative studies on bitter taste receptor evolution have primarily focused on the relation to feeding behavior and taste perception, T2Rs also play crucial roles in the chemosensory cells of various extra-oral organs, including the brain, airways, and gastrointestinal and reproductive organs. While the function of these ectopic taste receptors has been intensively studied in mammalian models, the evolutionary origins and conservation of each ectopic function remain poorly understood. These functions may also contribute to the T2R evolution in both aspects of gene number and receptive ranges of substances, hence the comparative evolutionary study on such ectopic functions using a variety of species can shed light on the deeper mechanisms of bitter taste receptor evolution.

## Supplementary Materials

The following supporting information can be downloaded at: https://www.mdpi.com/article/10.3390/ijms252312654/s1.

## Abbreviations

T2R	Taste receptor type 2 (bitter taste receptor) protein
*Tas2r/TAS2R*	Taste receptor type 2 (bitter taste receptor) gene
T1R	Taste receptor type 1 (sweet/umami taste receptor) protein
*Tas1r/TAS1R*	Taste receptor type 1 (sweet/umami taste receptor) gene
OR	Olfactory receptor (protein in upright or gene in italic)
TAAR	Trace amine-associated receptor (protein in upright or gene in italic)
V1R	Vomeronasal receptor type 1 (protein in upright or gene in italic)
V2R	Vomeronasal receptor type 2 (protein in upright or gene in italic)
GPCR	G protein-coupled receptor
TM	Transmembrane
QSM	Quorum-sensing molecule
EEC	Enteroendocrine cell
